# Evaluation of antigenic differences between wild and Sabin vaccine strains of poliovirus using the pseudovirus neutralization test

**DOI:** 10.1038/s41598-019-48534-1

**Published:** 2019-08-19

**Authors:** Minetaro Arita, Masae Iwai-Itamochi

**Affiliations:** 1 Department of Virology II, National Institute of Infectious Diseases, 4-7-1 Gakuen, Musashimurayama-shi, Tokyo 208-0011 Japan; 2 Department of Virology, Toyama Institute of Health, 17-1 Nakataikoyama, Imizu-shi, Toyama 939-0363 Japan

**Keywords:** Clinical microbiology, Applied microbiology

## Abstract

In the endgame of global polio eradication, serosurveillance is essential to monitor each country’s vulnerability to poliomyelitis outbreaks. Previously, we developed pseudovirus poliovirus (PV) neutralization test (pPNT) with type 1, 2, and 3 PV pseudovirus (PV_pv_), which possess a luciferase-encoding PV replicon in the capsids of wild-type strains (PV_pv_[WT]), showing that pPNT with type 2 and 3 PV_pv_(WT) but not type 1 shows high correlation with the conventional PV neutralization test (cPNT) performed with vaccine strains. Here, we analyse the antigenicity of PV_pv_(WT) and PV_pv_ with capsid proteins of Sabin vaccine strains (PV_pv_[Sabin]) in human serum. Type 2 and 3 PV_pv_(WT) and PV_pv_(Sabin) show similar antigenicity in the analysed set of human sera in contrast to type 1 PV_pv_. The levels of PV_pv_(Sabin) infection (%), including about 70% of PV_pv_ infection (%) measured in the presence of human serum diluted to the cPNT titre, serve as the optimal threshold values for pPNT (5% for type 1 and 2, 10% for type 3) to show high correlation with cPNT results. Our results suggest that pPNT with PV_pv_(Sabin) could serve as an alternative to cPNT and provide a rationale for pPNT threshold values.

## Introduction

Poliovirus (PV) is a small non-enveloped virus with a positive-sense single-stranded RNA genome of about 7500 nt belonging to the genus *Enterovirus*, the family *Picornaviridae*. After feco-oral infection, PV invades the central nervous system primarily via viremia, showing a positive correlation with PV paralytic rate^1^ and destroying motor neurons to cause poliomyelitis^2,3^. Immunization with live oral PV vaccine (OPV) or inactivated PV vaccine (IPV) induces production of anti-PV antibody in serum, which prevents PV central nervous system invasion and poliomyelitis.

Serosurveillance is performed to monitor anti-PV neutralization antibody titre in serum of potentially susceptible populations and to evaluate a country’s potential vulnerability a poliomyelitis outbreak. A conventional PV neutralization test (cPNT) using cell culture and infectious PV strains, which are usually attenuated vaccine strains, is currently performed to measure the anti-PV neutralization antibody^4^. Serosurveillance in Japan has been performed in prefectural laboratories every 2 to 3 years since 1974 (for about 1,100 to 1,800 individuals in 6 to 8 prefectures) in a wide age range (age of 0 to >40 year) to monitor vulnerability^5^. Serosurveillance is also important in Japan after the introduction of Sabin strain-based IPV (sIPV) after 2012^6^. sIPV induces similar persistence of anti-PV antibody to conventional wild-type-stain-based IPV when used as a booster in adults with history of OPV administration^7^. Recent reports suggest rapid declines of the antibody titre after sIPV administration^8–10^. The persistence of anti-PV antibody in children vaccinated with sIPV without OPV remains is poorly understood.

In the endgame of polio eradication, a high level of PV biosecurity is more important than ever. The third edition of the WHO Global Action Plan to minimize poliovirus facility-associated risk after type-specific eradication of wild polioviruses and sequential cessation of oral polio vaccine use (GAP III)^11^ was adopted by the WHO in May 2015 before indigenous wild type 2 PV was declared eradicated. GAPIII raises the biosafety level for type 2 PV strains (Annex 2 and 3 in GAP III), corresponding to BSL3 plus additional conditions and restricts the use of type 2 strains in biological tests in laboratories with conventional biosecurity levels.

One of the major challenges in implementing GAP III is the sustainability of the serosurveillance system under restricted use of infectious PV strains. After implementation, cPNT could not be performed in laboratories with conventional biosecurity levels. We previously produced a virus-free PV pseudovirus (PV_pv_)^12^ that could serve as an alternative to infectious PV. PV_pv_ possesses a luciferase-encoding PV replicon in the capsid and shows single cycle infection in susceptible cells (adsorption/uncoating/replication) without producing an infectious virus. PV_pv_ has been used for quantitative analyses of antiviral effects^13^ and anti-type 2 PV neutralization activity in stool in the context of infection^14^.

Neutralization tests with pseudotyped viruses have been developed for viruses under the control of high biosecurity levels (e.g. Ebola virus, SARS-CoV, lyssaviruses, Nipah virus, highly pathogenic avian influenza A viruses)^15–20^. In a previous study, we developed a pseudovirus PV neutralization test (pPNT) using PV_pv_ with the capsids of wild-type strains^21^. Following our report, other groups have also found good correlations between pPNT and cPNT^22,23^.

pPNT is largely characterized by three factors: (1) capsid protein type (*e*.*g*. capsid proteins of wild-type strains or vaccine strains), (2) titre or amount of PV_pv_, and (3) threshold values of PV_pv_ infection to determine the neutralizing antibody titre. The capsid protein type is critical for type 1 PV, exemplified by significant antigenic differences between the type 1 Sabin strain and the parental Mahoney strain by the VP3-T60K mutation^24,25^, and by an emergence of the type 1 wild-type strain with significantly low antigenicity^26^. Absolute titre or infectious units of PV_pv_ could be determined using focus assay^12^. The luciferase signal of PV_pv_-infected cells might be useful to determine the relative titre^22^. In cPNT, neutralization titre is determined by an endpoint reading for the cytopathic effect on infected cells, so it is an all-or-none reading. In pPNT, neutralization titre is determined by reading the PV_pv_ infection, which represents a percentage of luciferase signal in the infected cells, which is not an all-or-none reading. Therefore, threshold values are required for pPNT to define apparent “neutralization” of PV_pv_. Threshold values of PV_pv_ infection have been inductively estimated to give the best fit to cPNT results (2.5% to 25% of PV_pv_ infection)^21,22^. Variation of reported threshold values might suggest robustness of pPNT. Seropositive rates determined at a wide range of threshold values were substantially unaffected (*e*.*g*., seropositive rates for type 2 were 92% to 95% determined in a range of threshold values of 2.5% to 10%)^21^. The rationale to determine the threshold values remains to be provided.

Here, we analyse the antigenicity of PV_pv_ with capsid proteins of wild-type strains (PV_pv_[WT]) or of Sabin strains (PV_pv_[Sabin]), in a set of human sera obtained from healthy volunteers (ages of 1 to 76, total 131 samples). In addition, we provide a rationale for the threshold values for pPNT based on the distribution of PV_pv_ infection at cPNT titre.

## Results and Discussion

### Development of PV_pv_ with Sabin strain capsid proteins

**Figure 1:**
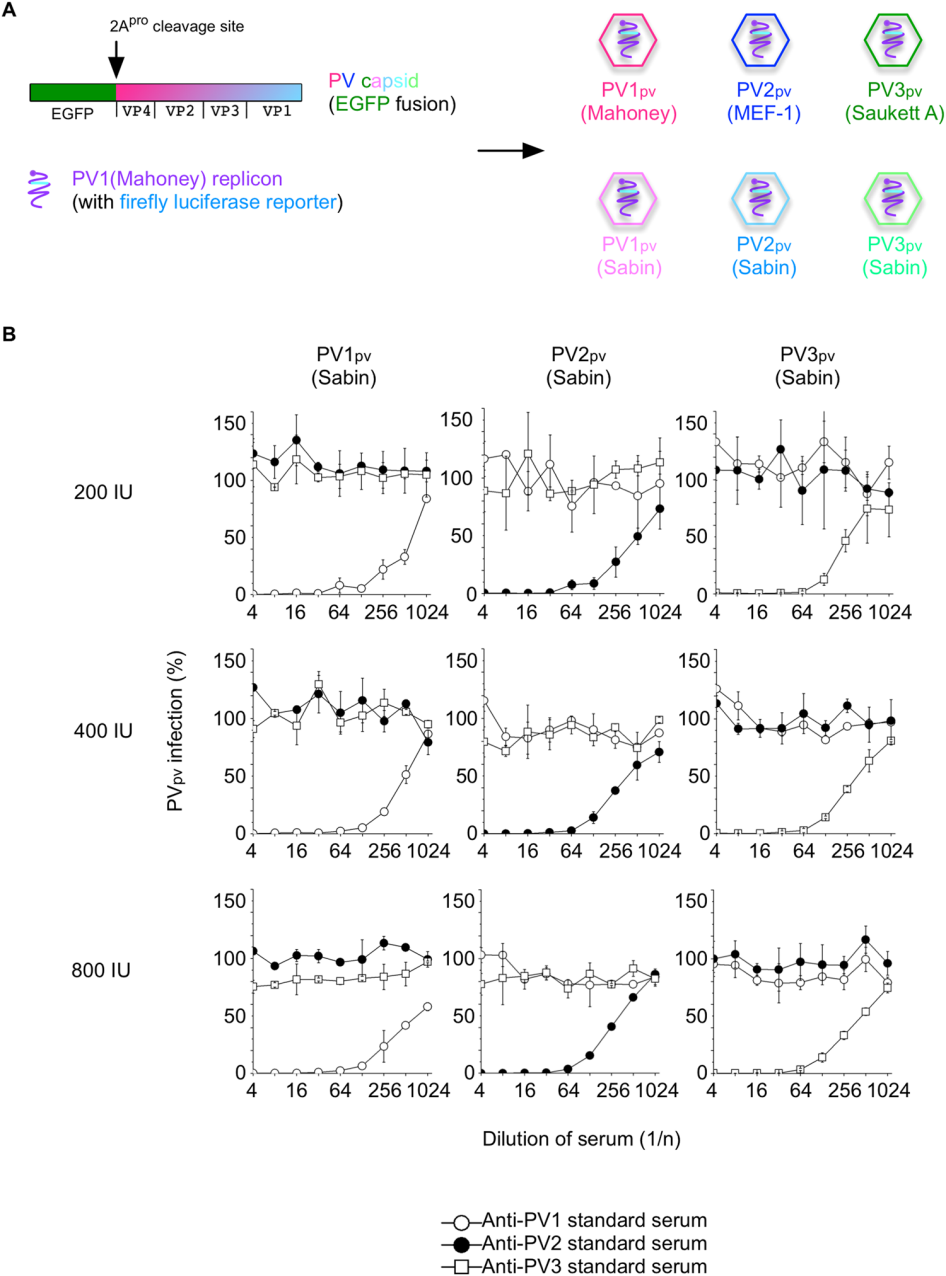
Optimization of the PV_pv_(Sabin) amounts in pPNT. (**A**) Schematic view of PV capsid, replicon, and PV_pv_ used in this study. (**B**) Neutralization curves of PV_pv_(Sabin) (200, 400, or 800 IU) with standard anti-PV sera (128 U per 50 μL) are shown. PV_pv_ infection in the absence of human serum is taken as 100%.

In a previous study, we produced type 1, 2, and 3 PV_pv_, which possess a luciferase-encoding PV replicon based on the Mahoney strain in the capsids of wild-type strains (type 1 Mahoney, type 2 MEF-1, and type 3 Saukett A in PV1 _pv_[WT], PV2 _pv_[WT], and PV3_pv_[WT], respectively)^12,21^. Here, we constructed PV capsid expression vectors that express the capsid proteins of type 1, 2, and 3 Sabin strains. We also produced type 1, 2 and 3 PV_pv_, which possess a luciferase-encoding PV replicon based on the Mahoney strain in the capsids of type 1, 2, and 3 Sabin strains, respectively (PV1 _pv_[Sabin], PV2 _pv_[Sabin], and PV3_pv_[Sabin], respectively) (Fig. 1A). We obtained type 1, 2, and 3 PV_pv_(Sabin) with high titres (2.5 × 10^7^, 1.2 × 10^7^ IU, and 6.0 × 10^6^ IU per mL, respectively), which were about 10-fold lower than PV_pv_(WT)^12,21^. Next, we optimized the amounts of PV_pv_(Sabin) for pPNT with standard antisera (Fig. 1B). Specific and similar neutralization profiles were observed for PV_pv_(Sabin) at a range of the examined amounts (200, 400, or 800 IU)^21^. We used 400 IU of PV_pv_(Sabin) in pPNT.

### Evaluation of PV_pv_ infection in the presence of human serum diluted to the cPNT titre

**Figure 2:**
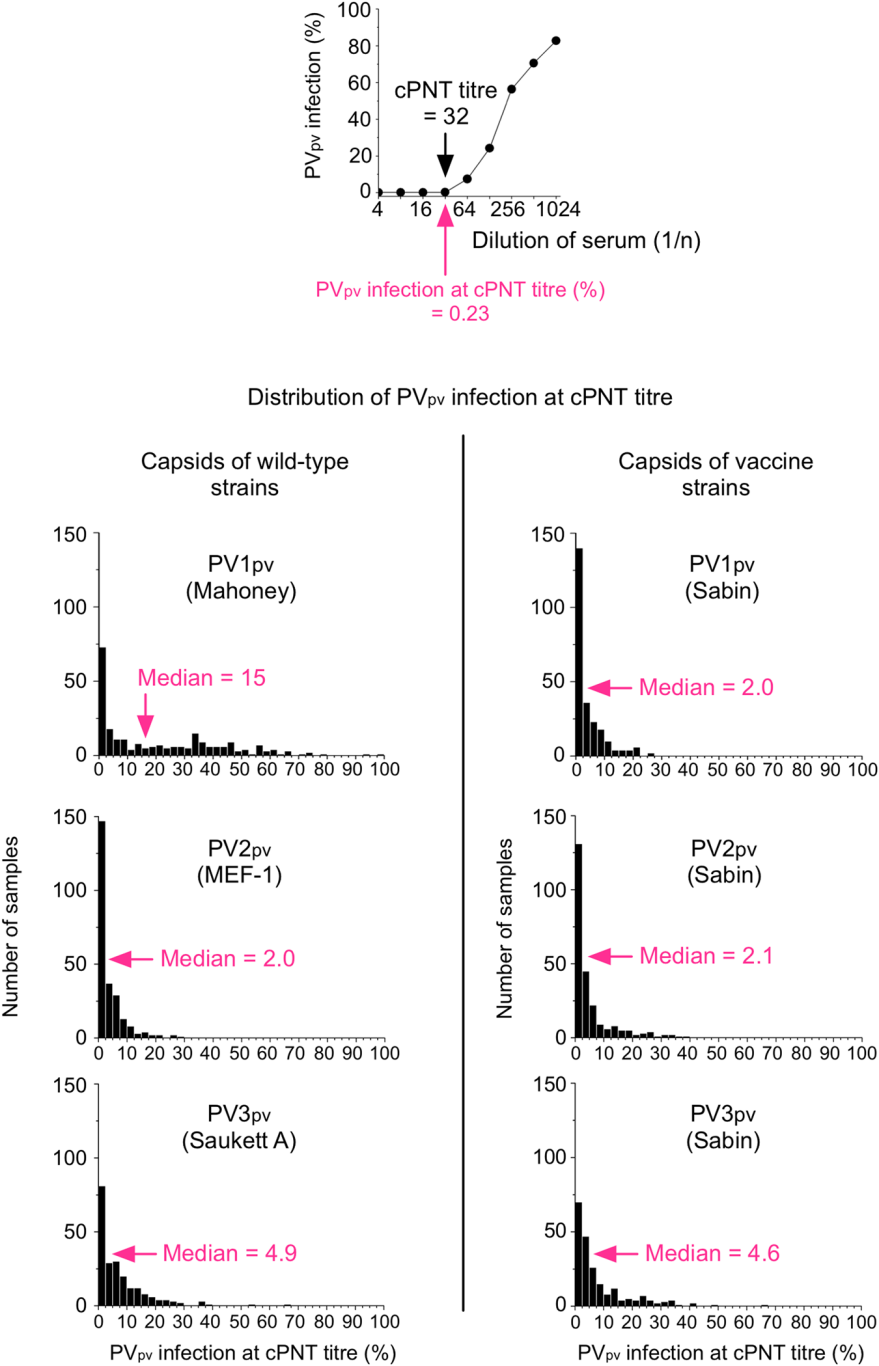
Comparison of neutralization profile of PV_pv_ (WT) and PV_pv_(Sabin) in human serum. Upper panel: An example of a PV_pv_ neutralization curve with a human serum sample with a cPNT titre of 32. PV_pv_ infection (%) at the cPNT titre is shown. PV_pv_ infection in the absence of human serum is taken as 100%. Lower panel: Distribution of PV_pv_ infection (%) at the cPNT titre. Results of two independent experiments with a set of human sera (total 131 samples) are shown.

**Figure 3:**
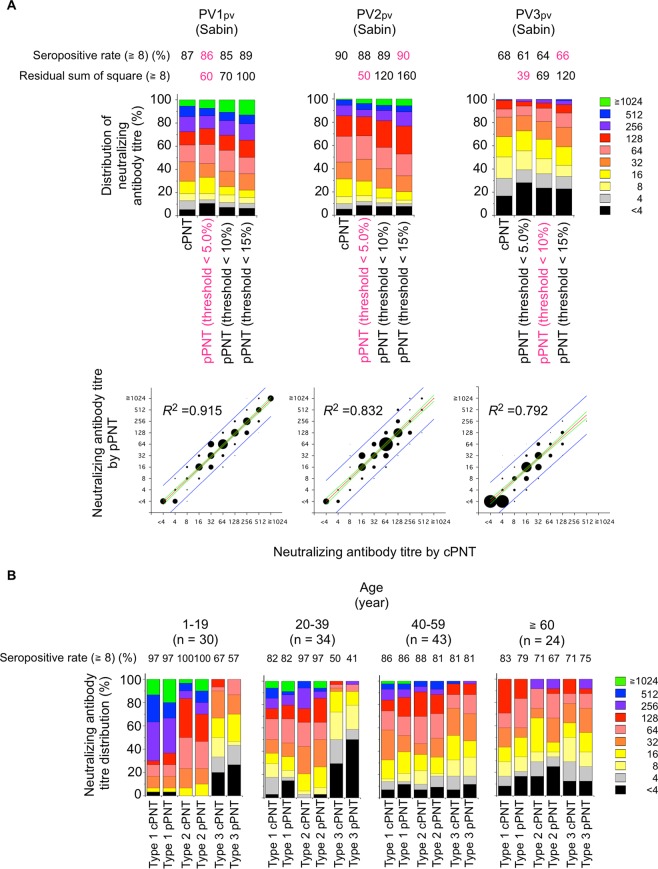
Determination of the threshold values for pPNT. (**A**) Upper panels: Comparison of cPNT and pPNT. Distribution of neutralizing antibody titre in human serum samples (n = 131) determined by cPNT or pPNT with a range of threshold values are shown. Optimal conditions are highlighted in magenta. Lower panels: scatter plot of neutralizing antibody titre determined by cPNT and pPNT. Threshold values for pPNT are 5% for type 1 and 2 or 10% for type 3. The numbers of serum samples at the corresponding spots are visualized by the size of circles. The regression line (red), 95% confidence interval (green), and 95% prediction interval (blue) are shown. (**B**) Distributions of neutralizing antibody titre obtained by cPNT and pPNT in different age groups. Threshold values for pPNT are 5% for type 1 and 2 or 10% for type 3.

Previously, we performed pPNT with PV_pv_(WT) for 131 human serum samples^21^. We determined the threshold values by maximizing the fitness of the seropositive rates (population with neutralization titre of at least 8). However, we could not directly compare the results because cPNT was performed with Sabin strains. To analyse the direct correlation between cPNT and pPNT, we analysed the distribution of PV_pv_ infection (%) in the presence of human serum diluted to the cPNT titre (Fig. 2). The PV_pv_(Sabin) infection showed a sharp peak around 0%, with medians of 2.0%, 2.1%, and 4.6%, for type 1, 2, and 3 PV_pv_(Sabin), respectively. The percentages of the serum samples that showed less than 5% PV_pv_ infection at the cPNT titre were 71%, 71%, and 54% for type 1, 2, and 3 PV_pv_(Sabin), respectively. The percentages of the serum samples that showed less than 10% PV_pv_ infection at the cPNT titre were 88%, 84%, and 72% for type 1, 2, and 3 PV_pv_(Sabin), respectively. A broader peak width for type 3 than types 1 and 2 might reflect intrinsic weak antigenicity of type 3 PV in humans vaccinated with OPV (Fig. 3). The profile of the curves of type 2 and 3 PV_pv_(WT) were like type 2 and 3 PV_pv_(Sabin)^21^. In contrast, the curve for PV1_pv_(WT) was significantly different from PV1_pv_(Sabin), and it showed a broad peak width (median of 15%). These results indicated substantial suppression of PV_pv_ infection in the presence of human serum diluted to the cPNT titre and significant antigenic differences for type 1 PV_pv_.

### Threshold values for PV_pv_(Sabin)

Next, threshold values for PV_pv_(Sabin) were analysed using the seropositive rate and the degree of correlation to compare to cPNT (Fig. 3A). For type 1 and 2 PV_pv_(Sabin), the seropositive rates were almost equal at a range of examined threshold values (5% to 15% of threshold values) and within 10% of variation from cPNT. PV3_pv_(Sabin) showed relatively low seropositive rate (61%) with a threshold value of 5% (Fig. 2). The highest degree of correlation between cPNT and pPNT in terms of residual sum of square was observed for threshold values of 5%. This suggested that threshold values of 5% might be optimal for pPNT with type 1 and 2 PV_pv_(Sabin). For PV3_pv_(Sabin), a threshold value of 10% seemed to give an optimal correlation. This suggests that the levels of PV_pv_ infection, under which about 70% of the distribution was included around the peak, serves as the optimal threshold for pPNT (Fig. 2). With threshold values of 5% for type 1 and 2 PV_pv_(Sabin) and 10% for PV3_pv_(Sabin), pPNT with PV_pv_(Sabin) showed high correlation with cPNT (*R*2 of 0.92, 0.83, and 0.79, respectively). For type 2 and 3, the results of PV_pv_(Sabin) were like PV_pv_(WT) (*R*2 of 0.81 and 0.77, respectively). For type 1, the correlation degree was drastically improved with PV_pv_(Sabin) compared to PV_pv_(WT) (*R*2 of 0.92 vs. 0.59), and the correlation between cPNT and pPNT was consistently observed among different age groups with PV_pv_(Sabin) in contrast to PV_pv_(WT) (Fig. 3B)^21^. For type 2 and 3 PV_pv_(Sabin), the correlation was also observed among the age groups, as previously observed for PV_pv_(WT)^21^. One of the critical points in PV serosurveillance is in the estimation of seropositive rate. Accurate determination of overall and age-specific seropositive rates by pPNT supports the potential of pPNT as an alternative to cPNT along with the observed high correlation.

### Reproducibility of pPNT

**Figure 4:**
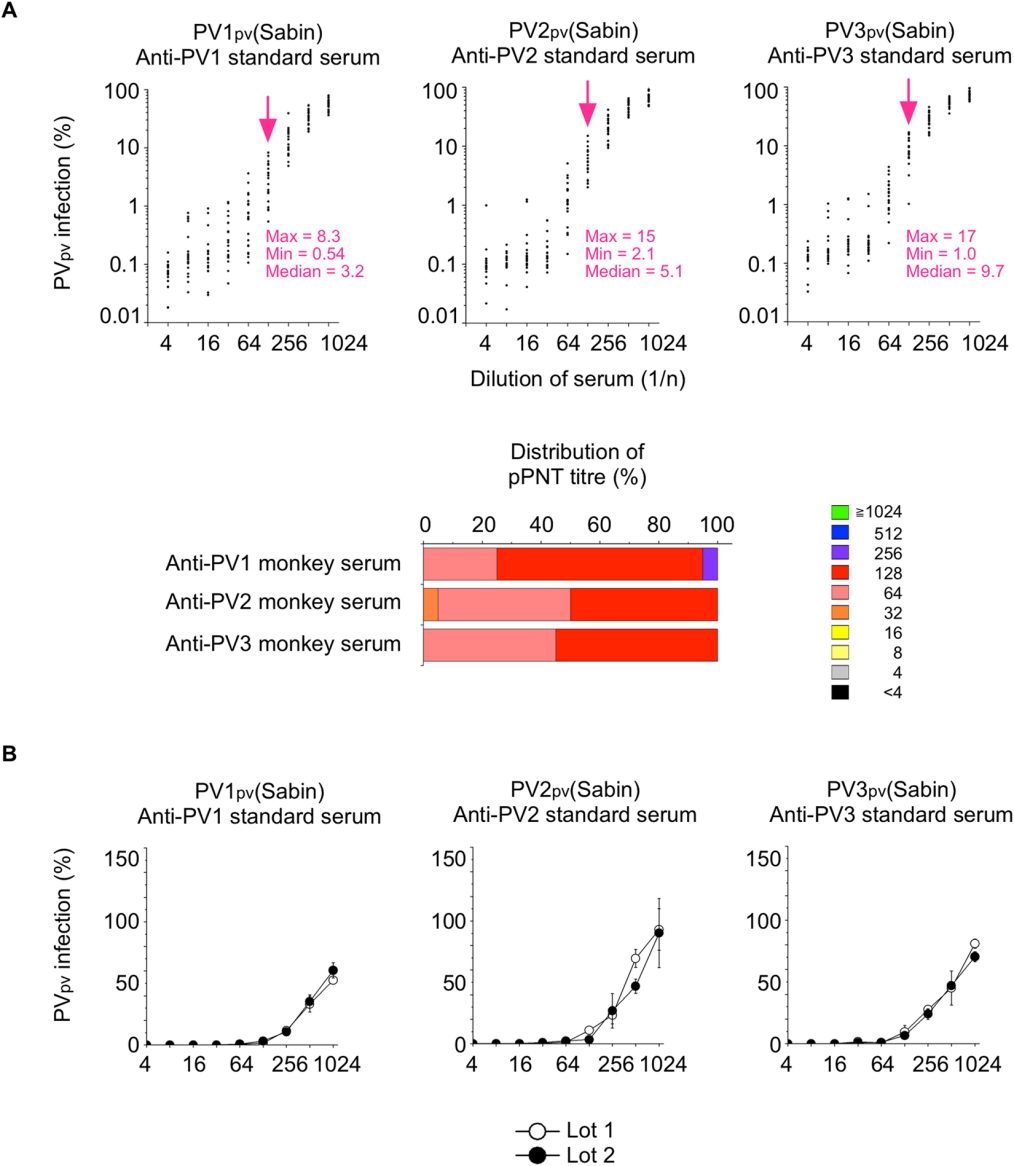
Reproducibility of pPNT with standard monkey anti-PV sera. (**A**) Test with standard monkey anti-PV sera (128 U per 50 μL). PV_pv_ infection in the absence of standard serum is taken as 100%. Results of twenty independent experiments are shown. Lower panel: Distribution of pPNT titre for standard anti-PV sera. Threshold values for pPNT are 5% for type 1 and 2 or 10% for type 3. (**B**) Test with different lots of PV_pv_(Sabin). Neutralization curve of two different lots of PV_pv_(Sabin) with standard monkey anti-PV sera is shown.

**Figure 5:**
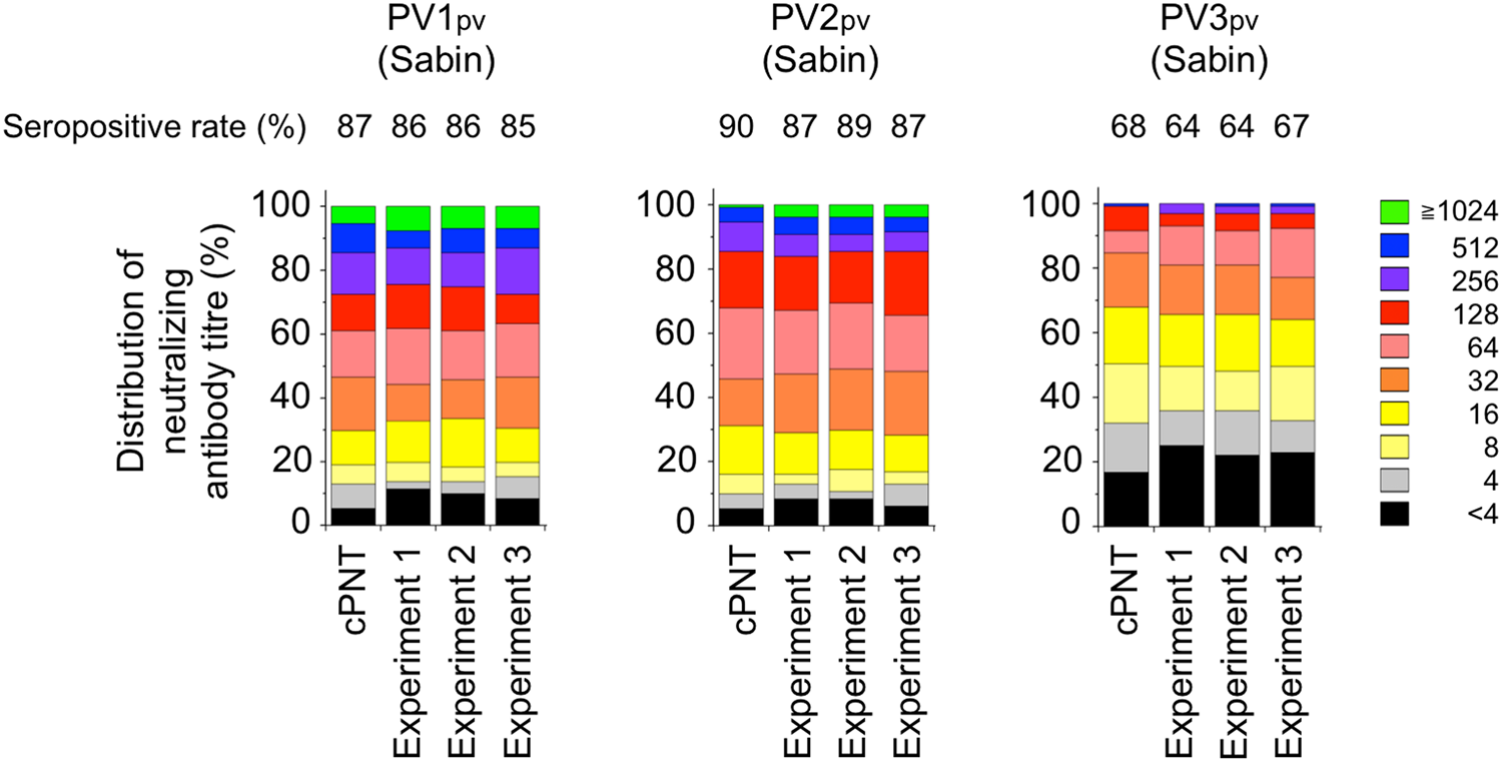
Reproducibility of pPNT with human sera. Distributions of neutralizing antibody titre in human serum samples (n = 131) determined by cPNT or pPNT (three independent experiments) are shown. Threshold values for pPNT are 5% for type 1 and 2 or 10% for type 3.

We analysed the reproducibility of pPNT (Fig. 4). First, we tested the reproducibility of pPNT with PV_pv_(Sabin) using standard monkey anti-PV sera (128 U per 50 μL). In twenty independent experiments, median values of PV_pv_(Sabin) infection at the cPNT titres of standard antisera appeared like the threshold values determined for each type (medians of 3.2%, 5.1%, and 9.7% for type 1, 2, and 3 PV_pv_[Sabin] respectively). In 19/20 of the experiments, the pPNT titres of standard sera were determined as 64 or 128 (Fig. 4A). We also analysed lot-to-lot differences of PV_pv_(Sabin) with the standard monkey anti-PV sera (Fig. 4B). Two different lots of PV_pv_(Sabin) showed similar neutralization curves with the antisera. Next, we tested the reproducibility with a set of human sera (total 131 samples) in three independent experiments (Fig. 5, Supplementary Data 1). We found consistent seropositive rates and distribution of neutralization titre among the experiments. These results suggest high reproducibility of pPNT and robustness of established conditions.

**Figure 6:**
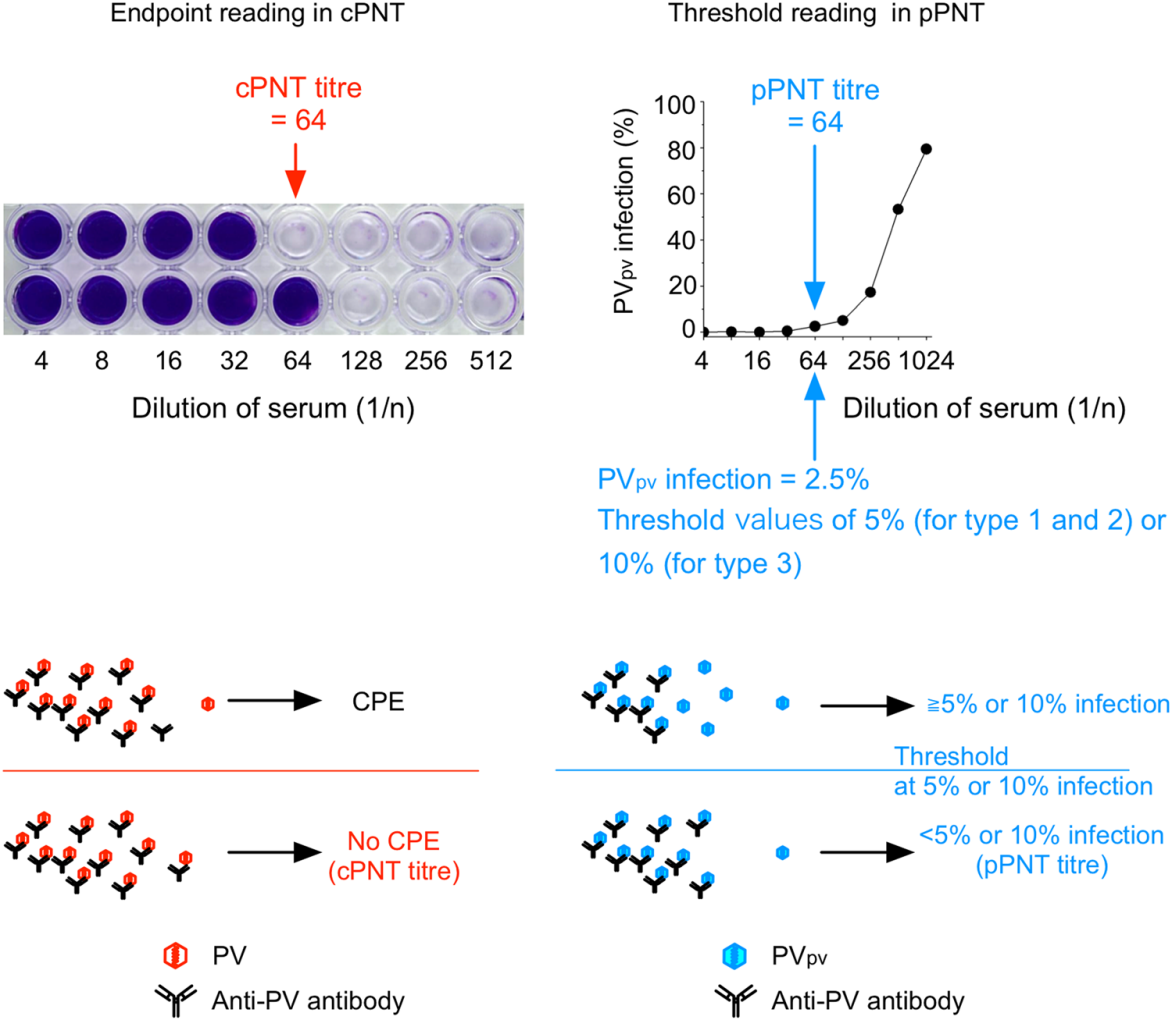
Principles of cPNT and pPNT. Upper panels: Raw data of cPNT and pPNT. Titre determined by cPNT or pPNT are shown. Lower panels: Schematic presentation of PV neutralization in cPNT and pPNT.

In summary, we clarified antigenic differences for type 1 PV strains using pPNT, and we provided a rationale for determining the threshold values for pPNT. With high reproducibility and safety without using infectious viruses, pPNT with PV_pv_(Sabin) could serve as an alternative to cPNT in serosurveillance in the endgame of the polio eradication program (Fig. 6).

## Methods

### Cells, viruses, and human sera

RD cells (human rhabdomyosarcoma cells) and HEK293 cells (human embryonic kidney cells) were cultured as monolayers in Dulbecco’s modified Eagle medium (DMEM) supplemented with 10% foetal calf serum (FCS). Vero cells (African green monkey kidney cells) were cultured as monolayers in Eagle’s Minimum Essential Medium (EMEM) supplemented with 0.11% bovine serum albumin (BSA) (fraction V, Sigma). RD cells were used for PV and pPNT titration. Vero cells were used for cPNT. HEK293 cells were used to produce PV_pv_. Type 1, 2, and 3 PV_pv_(WT) possess a luciferase-encoding PV replicon based on the Mahoney strain in capsid proteins derived from PV1(Mahoney), PV2(MEF-1), and PV3(Saukett A), respectively^12,21^. Human sera were collected from healthy volunteers (ages of 1 to 76) under informed consent from themselves or a parent or legal guardian for minors. Experiments performed here were approved by the Committee for Ethical Regulation of the National Institute of Infectious Diseases, Japan. All experiments were performed in accordance with relevant guidelines and regulations.

### General methods of molecular cloning

*Escherichia coli* strain XL10gold (Stratagene) was used to prepare plasmids. Ligation of DNA fragments was performed using an In-Fusion HD Cloning Kit (Clontech). PCR was performed using KOD Plus DNA polymerase (Toyobo). Reverse transcription-PCR (RT-PCR) was performed using a ReverTra -Plus- kit (Toyobo). DNA sequencing was performed using a BigDye Terminator v3.0 cycle sequencing ready reaction kit (Applied Biosystems) and analysed with a 3130 genetic analyser (Applied Biosystems).

### Construction of expression vectors for the capsid proteins of type 1, 2, or 3 Sabin strains

To construct expression vectors of capsid proteins of type 1, 2, and 3 Sabin strains (GenBank: AY184219, AY184220, and AY184221, respectively), the enhanced green fluorescence protein (EGFP) gene was fused to capsid-protein-coding regions of type 1, 2, and 3 Sabin strains using PCR before being inserted into pHEK293 Ultra Expression Vector I (TaKaRa) digested by *Sma*I and *Sal*I. EGFP coding regions were amplified using PCR with pIRES2-EGFP (Clontech) as the template and the following primer set (coding regions of EGFP with a linker in the primers are underlined): 5′TGCTTAAGCCTCCCCACCATGGGAGCTCTGAGCAAGGGCGAGGAG3′ 5′GTAGGTGGTCAGGCCCTTCTTGTACAGCTCGTCC3′. The PV capsid-protein-coding region was amplified using RT-PCR with viral genomic RNA as the template and the following primer sets (coding regions of the capsid proteins in the primers are underlined):

Type 1 Sabin:

5′GGCCTGACCACCTACGGTGCTCAGGTTTCATCACAGAAAGTGGGC3′

5′TGCCTGCAGGTCGACTTAATATGTGGTCAGATCCTTGGTGGAGAG3′

Type 2 Sabin:

5′GGCCTGACCACCTACGGCGCCCAAGTTTCATCACAGAAAGTTGG3′

5′TGCCTGCAGGTCGACTTAATAAGTCGTTAATCCCTTTTCTGGTAG3′

Type 3 Sabin:

5′GGCCTGACCACCTACGGAGCTCAAGTATCATCCCAAAAAGTAGGC3′

5′TGCCTGCAGGTCGACTTAATATGTGGTCAAACCTTTCTCAGATAA3′

### Preparation of PV_pv_

PV_pv_ was prepared as previously reported with modifications^12^. Briefly, a six-well plate (Falcon) with a 10% confluent monolayer of HEK293 cells was transfected with 2 μg of corresponding PV capsid-expression vectors per well using Lipofectamine 3000 reagent (Invitrogen). The cells were incubated at 35 °C in 2 ml DMEM supplemented with 10% FCS per well for 24 h. RNA transcripts of PV replicons were obtained using a RiboMAX large-scale RNA production system – T7 kit (Promega) with *Dra*I-linearized DNA of pPV-Fluc mc, which encodes a PV replicon based on PV1(Mahoney) that has firefly luciferase gene instead of the capsid-coding region as the template. RNA transcripts were transfected into the monolayer of HEK293 cells transiently expressing PV capsid proteins at 24 h post-transfection using Lipofectamine MessengerMAX reagent (Invitrogen). Cells were harvested at 24 h post-transfection of the RNA transcripts when most of the cells show CPE. The cells were then stored at **−**20 °C. The infectious unit of the PV_pv_ stock solution was determined by counting the number of HEp-2c cells infected with PV_pv_ 8 h post-infection (p.i.), which were stained using the 2C protein by indirect immunofluorescence as described previously^12^.

### cPNT

cPNT was performed according to the standard procedure recommended by the WHO with modifications^4^ as described previously^21^. Briefly, a 2-fold dilution series of human sera was prepared with EMEM supplemented with 0.11% BSA resulting in 1/4 to 1/1024 dilutions. 50 μL of diluted sera or EMEM supplemented with 0.11% BSA was added to three 96-well plates. 50 μL of type 1, 2, or 3 Sabin strains (100 50% cell culture infective dose (CCID_50_)) was added to each well of the plates (one plate for each serotype of PV with a total of 3 plates), and incubated at 37 °C 3 h. After incubation, 100 μL of Vero cell suspension in EMEM supplemented with 0.11% BSA (1.0 to 2.0 × 10^5^ cells) was added to each well of the plates, and the plates were incubated at 37 °C for 7 days. Neutralizing antibody titre of the serum was determined as 50% endpoints of the serum determined by the presence or absence of CPE in the cells.

### pPNT

pPNT was performed as reported previously with modifications^21^. 25 μL of human sera samples and standard anti-PV sera (128 U per 50 μL, positive control) were diluted with DMEM supplemented with 1% FCS by 4-fold (1/4 dilution). A 2-fold dilution series was made, resulting in 1/4 to 1/1024 dilutions. 5 μL of diluted sera or DMEM supplemented with 1% FCS (mock treatment) was added to three 384-well plates (Greiner Bio-One, 781080). Next, 5 μL of type 1, 2, or 3 PV_pv_ solution (400 IU) was added to each well of the plates (one plate for each serotype of PV_pv_ for a total of 3 plates). The plates were centrifuged (700 × *g*, 10 sec) (PlateSpin, KUBOTA). After centrifugation, plates were incubated at 4 °C overnight. After incubation, 20 μL of RD cell suspension in DMEM supplemented with 5% FCS (5.0 × 10^3^ cells) was added to each well of the plates. The plates were incubated at 37 °C for 7 h. Luciferase signal of the infected cells was measured at 7 h p.i. with Steady-Glo Luciferase Assay System (Promega) using a 2030 ARVO X luminometer (PerkinElmer) according to the manufacturer’s instructions. PV_pv_ infection was calculated as a percentage of luciferase signal of the infected cells. The luciferase signal in mock-treated cells was taken as 100%. Neutralizing antibody titre was determined as a reciprocal number of the highest dilution of the serum that suppressed PV_pv_ infection to less than 5%.

## Supplementary information

Dataset 1

## Data Availability

Raw data sets not included in the manuscript or in the supplementary information are available from the corresponding author upon request.
